# m6A Modification: A Double-Edged Sword in Tumor Development

**DOI:** 10.3389/fonc.2021.679367

**Published:** 2021-07-26

**Authors:** Runnan Gao, Mujie Ye, Baihui Liu, Meng Wei, Duan Ma, Kuiran Dong

**Affiliations:** ^1^ Department of Pediatric Surgery, Children’s Hospital of Fudan University, National Children’s Medical Center, Shanghai, China; ^2^ Key Laboratory of Metabolism and Molecular Medicine, Ministry of Education, Department of Bochemistry and Molecular Biology, Institute of Biomedical Sciences, Collaborative Innovation Center of Genetics and Development, School of Basic Medical Sciences, Fudan University, Shanghai, China

**Keywords:** epigenetics, genetics, m6A modification, tumor, therapeutic targets

## Abstract

Modification of m6A, as the most abundant mRNA modification, plays diverse roles in various biological processes in eukaryotes. Emerging evidence has revealed that m6A modification is closely associated with the activation and inhibition of tumor pathways, and it is significantly linked to the prognosis of cancer patients. Aberrant reduction or elevated expression of m6A regulators and of m6A itself have been identified in numerous tumors. In this review, we give a description of the dynamic properties of m6A modification regulators, such as methyltransferases, demethylases, and m6A binding proteins, and indicate the value of the balance between these proteins in regulating the expression of diverse genes and the underlying effects on cancer development. Furthermore, we summarize the “dual-edged weapon” role of RNA methylation in tumor progression and discuss that RNA methylation can not only result in tumorigenesis but also lead to suppression of tumor formation. In addition, we summarize the latest research progress on small-molecule targeting of m6A regulators to inhibit or activate m6A. These studies indicate that restoring the balance of m6A modification *via* targeting specific imbalanced regulators may be a novel anti-cancer strategy.

## Introduction

To date, various post-transcriptional RNA modifications have been identified in almost all living organisms (1). A number of studies have indicated that RNA modifications are of great importance in RNA epigenetics (2, 3). Among over 100 different modifications in RNA, N6-methyladenosine (m6A) was originally identified in the 1970s as the most abundant modification of mRNA and other noncoding RNAs in eukaryotes (4–8). With the rapid development of high-throughput assays, the formation, biological functions, and potential mechanisms of m6A modification have been gradually revealed.

m6A modification relies on a series of enzymes, which are considered “writers” (methyltransferases), “erasers” (demethylases), and “readers” (m6A-binding proteins) that can add, remove, or preferentially bind to m6A functional sites, thereby altering important biological functions, which is similar to DNA methylation (9, 10). Accumulating evidence has confirmed that m6A modification of RNA regulates the expression of target genes by affecting transcript splicing, transcript stability, translation efficiency, decay, and RNA-protein interactions, thus altering a series of malignant biological behaviors, such as cell proliferation, invasion, and metastasis (1, 9). Furthermore, several studies have indicated the promising roles of m6A RNA methylation in tumor initiation and development (11). Herein, we briefly introduce m6A modification regulators and describe in detail how m6A methylation functions as a double-edged sword to participate in the initiation and development of numerous tumors and the prospects of m6A modification as an important diagnostic biomarker and therapeutic target for tumor treatment.

## m6A Modifiation Machinery

**Figure 1:**
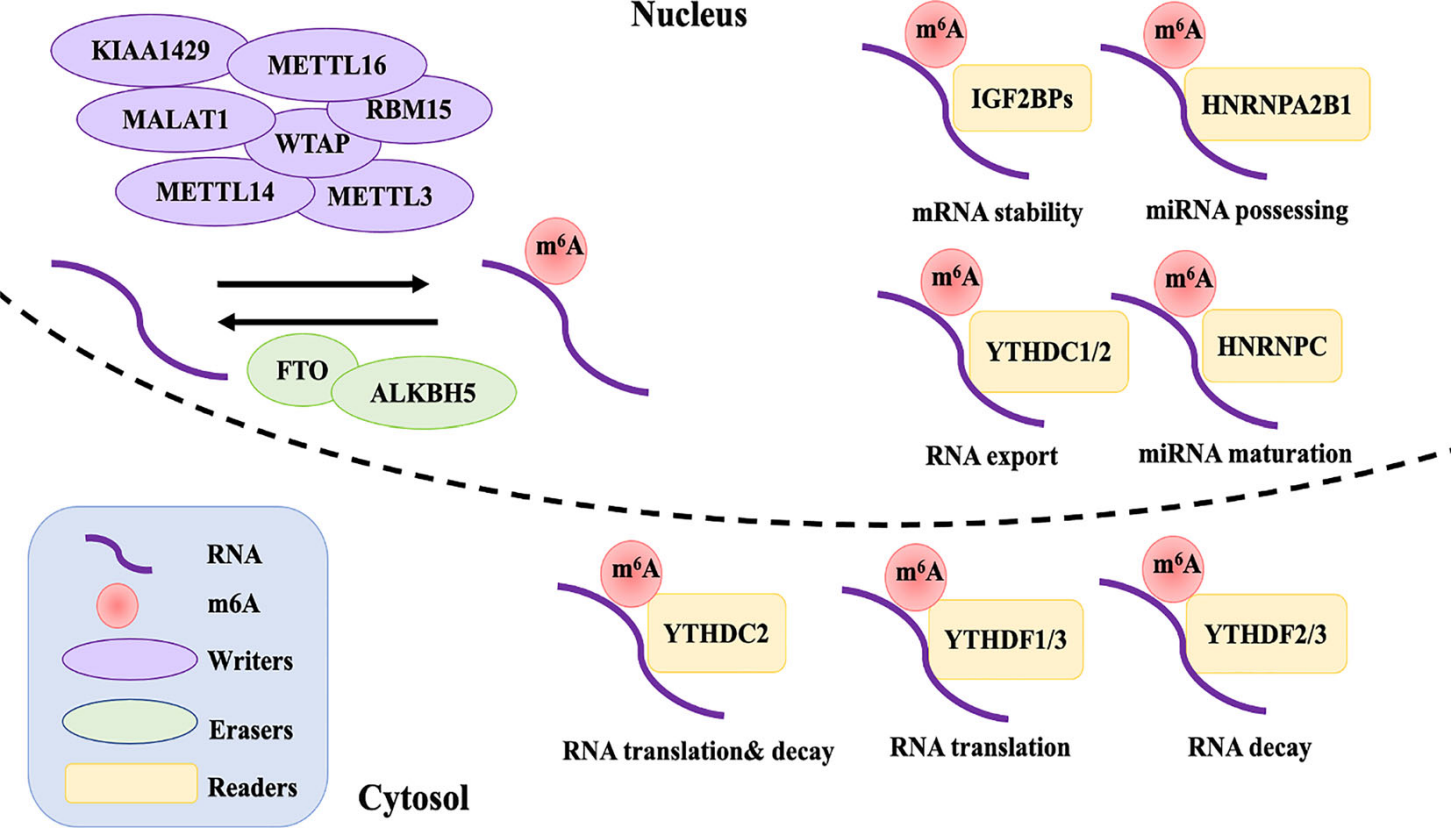
The reversible of m6A RNA modification is regulated by “writers”, “erasers”, and “readers”. Writers including METTL3, METTL14, WTAP, RMB15, KIAA1429, ZC3H13, and METTL16 interact with a special sequence of RRACH in mRNA that produces catalytic activity to add m6A methylation. The demethylations are performed by erasers FTO and ALKBH5. The differently biological functions of m6A in RNA metabolism are recognized by reader proteins from the YT521-B homology YTH domain (YTHDF1-3 and YTHDC1, 2), IGF2BPs (including IGF2BP1-3), and HNRNPC and HNRNPA2B1.

Given that m6A methylation is a reversible possess, the dynamic m6A landscape depends on the relative activity of writers and erasers, which fulfill their function by being recognized by readers (**Figure 1**). A change in charge, base pairing, and RNA-protein interactions comes with m6A-modified RNA, thus affecting the exporting, translocation, translation, and decay of RNA, impacting their ability to regulate gene expression (1).

### Writers

Currently, the process of m6A modification is thought to be catalyzed by a multi-component methyltransferase complex (12). Methyltransferase-like 3 (METTL3) was the first protein to be identified as an S-adenosylmethionine (SAM)-bound subunit complex that functions as a methyltransferase (13). Sharing 43% identity with METTL3, methyltransferase-like 14 (METTL14) and METTL3 form a stable dimer complex at a stoichiometric ratio of 1:1, which binds to Wilms’ tumor 1-associating protein (WTAP) and colocalizes in nuclear speckles (14, 15). Although METTL3 was identified as the catalytic core of the m6A complex, METTL14 has about 10 times the catalytic methylation activity of METTL3 (16). As for WTAP, it has no catalytic methylation activity, but acts as an adaptor protein that interacts with methyltransferases, affecting the methyltransferase activity of m6A and localization in nuclear speckles (15). However, studies have found that the influence of both METTL14 and WTAP on the total level of m6A is greater than that of METTL3 in HELA and 293FT cells (14). Other m6A methyltransferase components have subsequently been identified, including RNA binding motif protein 15 (RBM15), KIAA1429, zinc finger CCCH-type containing 13 (ZC3H13), and methyltransferase-like protein 16 (METTL16) (14, 17–19).

At present, understanding of m6A methyltransferase is exploratory, so it remains necessary to perform functional mining of known components and identify and screen for unknown components. Further research on these writers may provide novel biomarkers for tumor diagnosis and uncover new ideas for the discovery of many more effective tumor therapeutic targets.

### Erasers

So far, few proteins are known to remove m6A marks. Fat mass and obesity-associated (FTO) and alkB homologue 5 (ALKBH5) belong to the α-ketoglutarate-dependent dioxygenase family and have been identified as m6A demethylases, which may target different mRNAs (20, 21). Similar to various writers, overexpression or deletion of ALKBH5 or FTO alter the total level of m6A methylation in different cell lines (21, 22). Interestingly, alkB homologue 3 (ALKBH3), another reported eraser protein that exhibits demethylation activity, was recently found to interact more with m6A tRNA rather than mRNA or rRNA (23). However, the discovery of additional m6A demethylases and their functions and mechanisms are still under further study.

### Readers

Although the dynamic and reversible regulation of m6A modification is determined by both the writers and erasers of m6A, its biological functions also require various readers, which affect gene expression though functional interactions of downstream reader proteins (24). The reader proteins YTHDF1-3 and YTHDC1,2 from the YT521-B homology YTH domain family both have a conserved m6A-binding site, which directly reads and binds to the RRm6ACH consensus sequence of m6A-modified RNA (25). YTHDF2, the first m6A reader to be identified, recruits the CCR4-NOT deadenylase complex to promote the decay of targeted mRNAs (26). YTHDF1, on the other hand, improves the translation efficiency of m6A-modified mRNAs by coordinating with the translation initiation machinery (27). YTHDF3 seems to overlap with YTHDF1 and 2 in synergistically promoting methylated RNA translation through YTHDF1 and accelerates the decay of mRNA through direct interaction with YTHDF2 (28). YTHDC1 is an exclusively nuclear m6A reader protein, which can influence RNA export and regulate gene expression by interacting with transcripts and splice factors that contain m6A (29, 30). Although YTHDC2 is expressed in both the nucleus and cytoplasm, it is especially highly expressed in germ cells. It is considered to have a dual role, directly interacting with small ribosomal subunits to facilitate their translation and subsequently degrading them (31–33). Recently, reader proteins from a distinct family—insulin-like growth factor 2 mRNA binding proteins (IGF2BPs, including IGF2BP1-3)—have been reported to directly recognize m6A modification, thereby promoting the stability and storage of m6A-modified mRNA (34).

Except for directly recognizing the conserved m6A-binding domain, some reader proteins are suggested to influence the secondary structure of targeted mRNA and thereby indirectly modulate its abundance and splicing, which is termed the “m6A switch” (35). Heterogeneous nuclear ribonucleoprotein (HNRNP) proteins, HNRNPC and HNRNPA2B1, preferentially bind to mRNA possessing m6A loci, promoting primary miRNA processing and maturation by impacting splicing, export, and translation initiation (35, 36).

The potential number of m6A-modification readers that can be identified directly or indirectly is large, which includes a broad research space. Because m6A modifications rely on readers to perform their biological functions, the same m6A modifications may have opposite biological effects when combined with different readers. Thus, mRNA transcription can be altered differently by different groups of readers, and together, these groups affect cellular functions that can result in changes in physiological condition. When regulation goes wrong, it can contribute to the occurrence of various diseases, including tumors. Therefore, promoting or blocking the binding of m6A RNA to readers may become another means of tumor treatment in the future.

## The Dual Role Of m6A Modifications in Tumors

**Figure 2:**
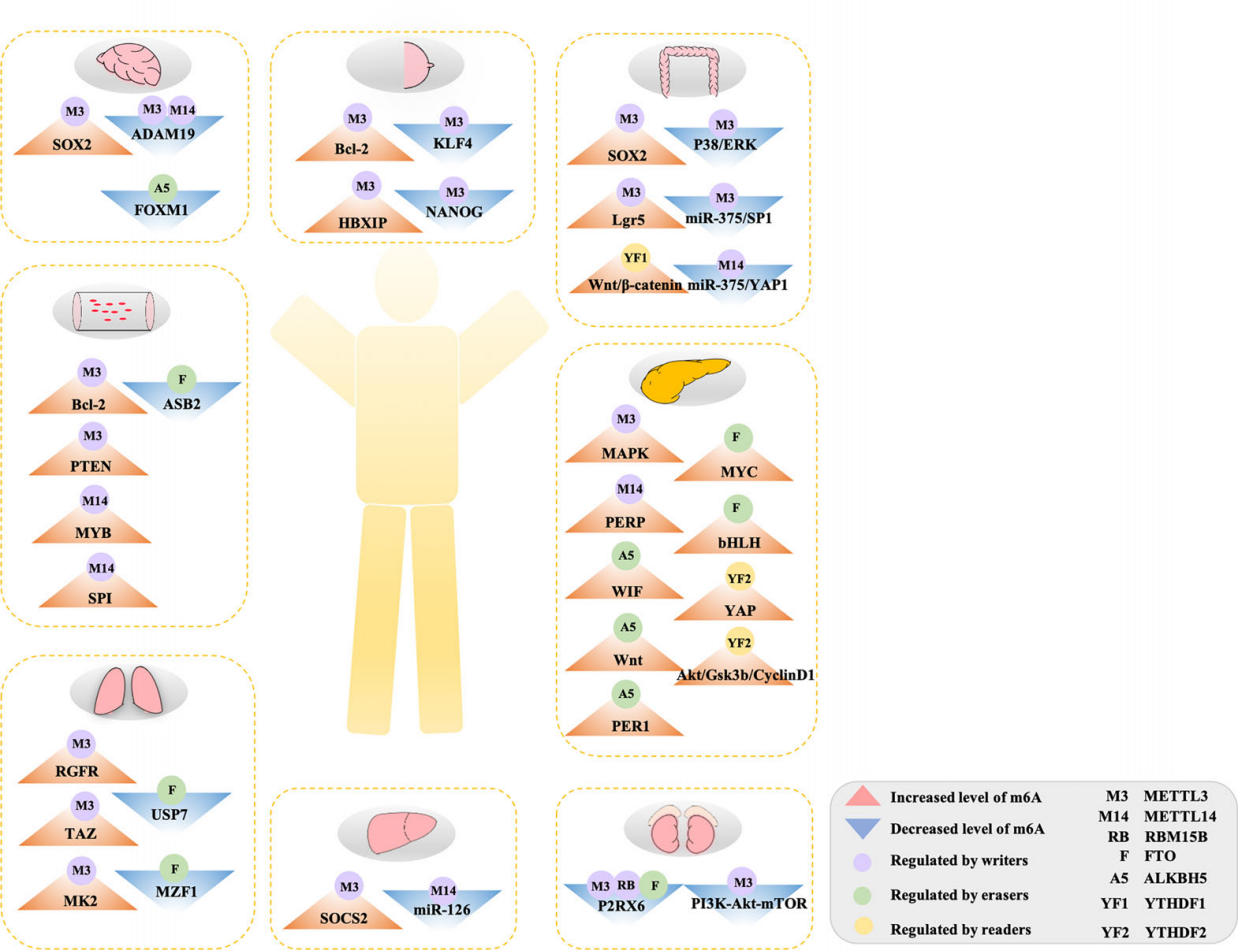
The double-edged role of m6A methylation in human cancers. Aberrant m6A modification regulated by diverse m6A-related regulators targeted on various genes or pathway to accelerate to initiation and development in tumors. See main text for more details.

Although it is believed that the impact of m6A on tumors is mainly through modulation of the expression of cancer-related genes, a variety of factors affect the target genes regulated by m6A on the progression of cancer during the regulation process (**Table 1**). Specifically, how m6A influences tumorigenesis by regulating targeted genes relies on three factors: (1) whether the targeted genes act as oncogenes or anti-oncogenes, (2) the levels of m6A in the cancer (depending on the activity or relative expression level of writers and/or erasers), and (3) the alteration of target mRNA after methylation (which relies on readers). Given these vital factors, the role of m6A in cancer is not easy to elucidate. In addition, there is growing evidence that m6A plays a dual role, which means that the same writer may contribute to tumorigenesis and suppress tumorigenesis in several cancers. Therefore, studying the mechanism of m6A modification and its post-modification regulation in different tumors (from solid to liquid) would be very valuable. In this review, we briefly summarize recent studies on m6A modification in a variety of tumors (**Figure 2**).

**Table 1:** List of m6A regulators involved in various tumors.

m6A regulators	m6A level	Tumor Type	Targets	Functions
METTL3↑	Up	Glioblastoma	Oncogene SOX2	METTL3 upregulate the expression of SOX2 (37)
		Breast cancer	Oncogene Bcl-2 HBXIP	METTL3 promote the expression of Bcl-2 (38) High levels of METTL3 enhance m6A modification of HBXIP, which accelerates HBXIP expression (39)
	
		Lung cancer	Oncogenes RGFR Oncogenes TAZ Oncogenes MK2 Suppressor SOCS2	METTL3 contributes to the several target oncogenes translation, as RGFR, TAZ, and MAPKAPK2 (MK2) (40–42) METTL3 accelerates the degradation of SOCS2 (43)
	
		Hepatocellular carcinoma		
		Colorectal cancer	Oncogene SOX2 LGR5	METTL3 increased expression of SOX2 and extend the decay of LGR5 (44, 45)
		Acute myeloid leukemia	Oncogenes c-MYC Oncogenes BCL2 Oncogenes PTEN	METTL3 contribute to the translation of targeted oncogenes c-MYC, BCL2, and PTEN (46, 47)
		Pancreatic cancer	MAPK Ubiquitin-dependent modification	METTL3 promotes chemo- and radioresistance which identified associating with mitogen- activated protein kinase (MAPK) cascades, ubiquitin-dependent modification in pancreatic cancer (48)
METTL3↓	Up	Glioblastoma	Oncogene ADAM19	Decreased METTL3 and/or METTL14 or increased FTO promotes the proliferation and self-renewal and decreases m6A levels on ADAM19 and enhances ADAM19 expression (11)
	Down	Breast cancer	KLF4 NANOG	ZNF217 interacts with METTL3 and inhibits the m6A methylation of KLF4 and NANOG, which ultimately leads to high expression of KLF4 and NANOG (49, 50)
		Colorectal cancer	p38/ERK pathway	Knockout in METTL3 and found it activated p-p38 and p-ERK (51)
		Renal cell carcinoma	PI3K-Akt-mTOR Pathway	METTL3 activate the pathway of PI3K-Akt-mTOR (52, 53)
METTL14↑	Up	Acute myeloid leukemia	MYB and MYC Oncogenes SPI	METTL14 targeted downstream genes MYB, MYC,SPI accelerate the growth of tumor (54, 55)
		Pancreatic cancer	PERP	METTL3 leads to upregulation of PERP (56, 57)
METTL14↓	Down	Glioblastoma	Oncogene ADAM19	Decreased METTL3 and/or METTL14 or increased FTO promotes the proliferation and self-renewal & decreases m6A levels on ADAM19 and enhances ADAM19 expression (37)
		Breast cancer	Unknown	
		Hepatocellular carcinoma	miR-126	METTL14 induce the interaction of miR126 with DGCR8 promoting tumorigenesis (58)
		Colorectal cancer	miR-375/YAP1 pathway miR-375/SP1 pathway	miR-375 targeted by METTL14 could suppress the growth of CRC through the miR-375/YAP1 pathway and inhibit the migration and invasion through the miR-375/SP1 pathway respectively (59)
		Renal cell carcinoma	P2RX6	Decreased expression of METTL14 and RBM15B, and increased FTO suppress the activation of P2RX6 (53)
WTAP↑	Up	Glioblastoma	Unknown	
		Acute myeloid leukemia	Unknown	
WTAP↓	Down	Breast cancer	Unknown	
KIAA1429↑	Up Down	Lung cancer Breast cancer	HOXA1 Unknown	KIAA1429 accelerated the gefitinib resistance of NSCLC by enhancing the stability of HOXA1 mRNA (60)
ZC3H13↓	Down	Breast cancer	Unknown	
RBM15B↓	Down	Renal cell carcinoma	P2RX6	Decreased expression of METTL14 and RBM15B, and increased FTO suppress the activation of P2RX6 (61)
FTO↑	Up	Pancreatic cancer	Oncogene MYC bHLH	FTO enhance the stability of proto-oncogene MYC and transcription factor bHLH (62)
FTO↑	Down	Glioblastoma	Unknown	
		Breast cancer	Unknown	
		Lung cancer	USP7 MZF1	FTO improve the stability of USP7 and enhance the expression of MZF1 (63, 64)
		Acute myeloid leukemia	Oncogenes ASB2	FTO promote the expression of oncogenes ASB2 (65)
		Renal cell carcinoma	P2RX6	Decreased expression of METTL14 and RBM15B, and increased FTO suppress the activation of P2RX6 (66)
ALKBH5↑	Up	Pancreatic cancer	Wnt pathway PER1	ALKBH5 decrease the expression of WIF protein and thus upregulate Wnt pathway; activate PER1 (57, 67)
ALKBH5↑	Down	Glioblastoma	Oncogene FOXM1	ALKBH5 promotes GSC proliferation and GBM progression by enhancing FOXM1expression through m6A modification (68)
		Breast cancer	Unknown	
		Acute myeloid leukemia	Unknown	
YTHDF1↑	Up	Colorectal cancer	WNT/β-catenin pathway	Downregulate the expression of YTHDF1 suppressed the tumorigenicity *via* the WNT/β-catenin pathway (69)
YTHDF1↑	Down	Breast cancer	Unknown	
YTHDF2↑	Up	Glioblastoma	Unknown IGF1/IGF1R	IGF1/IGF1R inhibitor targeting YTHDF2-expressing cells could suppress GSC viability and the growth of glioblastoma (70)
		Pancreatic cancer	YAP signaling Akt/GSK3b/CyclinD1 pathway	YTHDF2 inhibit adhesion and invasion through YAP signaling and accelerate the growth of tumor through Akt/GSK3b/CyclinD1 pathway (71)
YTHDF3↑	Down	Breast cancer	Unknown	
YTHDC3↑	Up	Colorectal cancer	Unknown	

### m6A Modification in Glioblastoma

Glioblastoma stem cells (GSCs) promote tumor growth and invasion, and their presence may predict a poor prognosis of glioblastoma (GBM), the most aggressive and lethal primary brain tumor (72). Multiple studies have shown that m6A modification plays a significant role in the tumorigenesis and development of GSCs. Decreased m6A level by knockdown of the expression of METTL3 and/or METTL14 or overexpression of the eraser FTO can significantly accelerate tumorigenesis, specifically promoting the growth of human GSCs, as well as their self-renewal and tumor progression. m6A sequencing further clarified that METTL3 or METTL14 knockout resulted in a decrease in the m6A enrichment level of mRNA and upregulates targeted oncogenes ADAM19 (11). Consistently, the elevated ALKBH5 expression associated with decreased global m6A level in GSCs has been suggested to contribute to their self-renewal and tumorigenesis. m6A-seq data identified that ALKBH5 interacts and co-localizes with FOXM1, which plays a necessary role in promoting tumorigenicity in GBM (68). These results demonstrate that tumorigenesis in GSC is negatively correlated with the general m6A modification level, indicating that the reduction of m6A RNA methylation, confirmed by either the knock-down of writers (METTL3/14) or overexpression of erasers (ALKBH5), significantly enhances the growth of tumors.

However, opposite results were reported in other studies. Although Visvanthan et al. further confirmed that METTL3 functions as a core regulator of m6A methylation in GSCs, interestingly, they identified a higher level of m6A and METTL3 in GSCs. The same group further found that the targeted mRNA oncogene SOX2 was upregulated by METTL3 in GSCs by altering radiation sensitivity and DNA repair efficiency (37). The oncogenic effect of METTL3 was also described by Li et al., with higher expression of METTL3 and YTHDF2 inducing extensive expression of mRNAs for splicing factors (73). Similarly, a study showed that m6A modification was often upregulated in GSCs compared with normal neural stem cells (NSC). Specifically, they found that an IGF1/IGF1R inhibitor targeting YTHDF2-expressing cells could suppress GSC viability and the growth of glioblastoma *in vivo* in an m6A-dependent manner (70). Another study found that WTAP was over-expressed in GBM patients, although it was classified as a tumor suppressor originally. However, whether the high expression of WTAP was through an m6A-dependent mechanism still remains unclear in GBM (74). These studies show that there is a higher level of m6A modification in GBM patients, by demonstrating that higher levels of writers (METTL3 or WTAP) and readers (YTHDF2) contribute to the progression of gliomas.

Given the essential function of m6A modification in GBM, studies have concentrated on whether the expression of m6A regulators has prognostic value. Chai et al. analyzed the correlation between 13 m6A modification-related genes with the clinicopathological features of 904 gliomas in databases (the Chinese Glioma Genome Atlas (CGGA, available online at www.cgga.org.cn, accessed on April 10, 2020) and The Cancer Genome Atlas (TCGA, available online at http://cancergenome.nih.gov, accessed on April 10, 2020) (73). Noticeably, they found that 11 of 13 m6A-related regulators were strongly linked to overall survival (OS), among which FTO was consistently a protective gene among all types of gliomas. In their study, they reported a seven-gene signature, including WTAP, RBM15, YTHDF2, YTHDF1, and ALKBH5 as risk genes, for their expression levels, which were positively associated with increasing malignancy of glioma, whereas FTO and YTHDC1 were negatively correlated with increasing malignancy and were therefore protective genes. Recently, another study also analyzed the correlation of m6A regulators and prognostic features and found that HNRNPC, WTAP, YTHDF1, and YTHDF2 were significantly upregulated in glioma tumors in patients with good OS (75). On the whole, the expression of related m6A regulators may be valuable for prognosis in glioblastoma.

### m6A Modification in Breast Cancer

Breast cancer (BC) is the most common malignancy and results in the largest number of deaths in women (76). Analyzing data from databases (the ONCOMINE and TCGA), researchers have found that m6A levels were decreased in 36 pairs of BC tissue compared to normal paired paracancerous tissue. Specifically, the level of m6A writers (METTL3, METTL14, and WTAP) decreased, whereas the erasers (FTO and ALKBH5) increased in BC tissue. Functionally, increasing global m6A levels dramatically inhibits human breast cell growth and metastasis, suppressing tumor progression, *via* overexpression of METTL14 and/or knockdown of the expression of the easer ALKBH5 (77). Studies further discovered a role of m6A methylases in BC; consistently, they also identified the writers ZC3H13 and METTL14, which functioned as tumor suppressor genes in BC patients using TCGA data (78). Similarly, by analyzing TCGA data, another study found that KIAA1429, YTHDF1, and YTHDF3 in BC tissues is upregulated, and the expression was closely linked to intrinsic subclasses and lymph node metastasis. Importantly, overexpression of YTHDF1 and YTHDF3 is significantly correlated in BC patients with poor prognosis (79). Several studies tried to further discover the mechanisms of these various m6A-related genes in the development of BC. Zhang et al. suggested that the activity of hypoxia-inducible factors (HIFs) in BC cell lines stimulated tumor progression by negative regulation of m6A methylation. Among them, ALKBH5-mediated demethylation in NANOG and KLF4 mRNA promotes tumorigenesis (49). Specifically, METTL3 interacted with the oncogene ZNF217 to suppress the m6A modification of NANOG and KLF4, which eventually led to upregulation of the mRNA expression of NANOG and KLF4 and accelerated tumorigenesis (50). These studies showed that low expression of writers (METTL3, METTL14, WTAP, and ZC3H13, but not KIAA1429) and high expression of erasers (FTO and ALKBH5) contributed to tumorigenesis in BC, suggesting a lower m6A modification in BC patients. Furthermore, the expression of readers (YTHDF1 and YTHDF3) may provide crucial prognostic tools for BC patients.

It is worth noting that these findings have been contradicted in other studies. For example, a study suggested that m6A level was increased in BC patients accompanied by up-regulated expression of METTL3. When METTL3 was knocked down, a decreased m6A level inhibited tumorigenesis, significantly reducing proliferation, accelerating apoptosis, and inhibiting the growth of tumors. Gene-specific m6A-qPCR further demonstrated that the oncogene Bcl-2 is a target of METTL3 in BC individuals (38). Consistently, a study showed that hepatitis B X-interacting protein (HBXIP) targeted by METTL3 has higher m6A levels in BC cells. In addition, they found that higher expression of METTL3 was not only found in clinical BC patients but also induces overexpression of HBXIP, which accelerates the proliferation of BC cells (39). Furthermore, one study found that MAGI3 with high levels of m6A methylation led to premature of polyadenylation, leading to this tumor suppressor gene to switch its function to promote tumor progression and ultimately facilitating the tumorigenesis of BC (80). Overall, abnormally elevated levels of m6A methylation at the target site can disrupt the normal physiological functions of RNA, thus accelerating the development of tumor formation.

### m6A Modification in Lung Cancer

Several studies have also reported that aberrant m6A methylation ultimately influences the progression of lung cancer, especially of non–small-cell lung carcinoma (NSCLC) (81). A report indicated that FTO plays a carcinogenic role in human NSCLC. The expression of FTO in both mRNA and protein levels was elevated in both NSCLC tissues and cell lines, accompanied by decreased m6A levels (63, 82). Further analysis of the mechanism showed that FTO downregulated the total level of m6A and subsequently improved ubiquitin-specific protease (USP7) mRNA stability mediated by the demethylase activity of FTO (63). Similarly, functional loss studies have shown that FTO knockdown can effectively inhibit lung cancer cell line proliferation and invasion. When FTO is overexpressed, the expression of MZF1 can be enhanced by decreasing the level of m6A and the MZF1 transcript stability, implementing oncogenic functions (64). Generally, the overexpression of FTO associated with decreased m6A promotes tumor formation in lung cancer.

In many studies, METTL3 provides a vital role in the growth, survival, migration, and invasion of lung cancer cell lines (40). Upregulation of METTL3 contributes to the translation of several target oncogenes, such as RGFR, TAZ, MAPKAPK2 (MK2), and DNMT3A, independent of its methyltransferase activity (40–42). A report also revealed a link between m6A modification and miRNA. High expression of METTL3 alleviated the suppression of lung cancer cell growth and migration caused by miR-338-5p (83). In addition, one study also investigated the post-translational modification of METTL3. They found that by modifying METTL3 with small ubiquitin-like modifier 1 (SUMO1), the m6A level was reduced on mRNAs, which eventually contributed to the progression of NSCLC (84). Tang et al. identified that KIAA1429 accelerated the gefitinib resistance of NSCLC *in vitro* and *in vivo* by enhancing the stability of HOXA1 mRNA in an m6A-dependent manner (60). Notably, these studies demonstrate that METTL3 may act in a tumorigenesis role in lung cancer *via* not only target mRNA and miRNA but also by being modified by other genes.

### m6A Modification in Hepatocellular Carcinoma

Hepatocellular carcinoma (HCC) is the main type of primary liver cancer, ranks as the fifth highest cause of mortality due to malignancy worldwide (85). Many studies have suggested that abnormal m6A methylation is linked to the development of liver cancer. In human HCC, high expression of METTL3 is strongly associated with elevated levels of m6A, which targets the tumor suppressor SOCS2 and eventually facilitates tumorigenesis. Furthermore, excessive m6A in SOCS2 is recognized by the reader YTHDF2, thereby accelerating the degradation of tumor suppressor, thus leading to the initiation of HCC (43). Therefore, upregulated METTL3 with an elevated m6A level inhibits the expression of tumor suppressors and contributes to the progression of HCC.

Likewise, modified expression of m6A shows an opposite trend during the progression of HCC. In HCC tissues, especially in tissues from metastatic HCC patients, METTL14 is considered to be the primary cause for the imbalanced m6A level. Reduced expression of METTL14 indicated poor prognosis for HCC patients, and its expression is even lower in metastatic HCC patients (58). The correlation between m6A modification and miRNA has also been demonstrated in HCC. However, one study noted that decreasing the expression of METTL14 reduced the level of m6A modification and microRNA126 (miR126). miR126 with low m6A modification was captured by DGCR8 and may eventually accelerate the progression of liver cancer (58). These studies all showed that the reduction of m6A modification is also significantly linked to the initiation and development of HCC.

### m6A Modification in Colorectal Cancer

Colorectal cancer (CRC) is a highly lethal form of cancer with high morbidity and mortality. Although there have been improvements in medical diagnosis and treatment, the global incidence of CRC continues to increase (86). METTL3 has been shown to serve as an oncogene in CRC in several studies. One study revealed that compared with normal tissues, the expression of METTL3 was higher in CRC, and with MeRIP-m6A-seq, they further noticed that the elevated m6A modification level increased the expression of SOX2 (which is required for stem-cell maintenance) *via* METTL3 interaction with IGF2BP2 to inhibit SOX2 degradation (44). In addition, another study found that METTL3 can also be recognized by IGF2BP1, prolonging the half-life of chromobox 8 (CBX8) mRNA, interacting with the leucine-rich repeat containing G-protein coupled receptor 5 (LGR5) promoter and maintaining H3K4me3 status. Altogether, these alterations contribute to the expression of LGR5, which eventually maintains the stemness of CRC cells and promotes drug resistance as well (45). Both the mRNA and protein levels of YTHDF1 were found to be highly expressed in CRC. Mechanistically, one study noticed that downregulated expression of YTHDF1 remarkably suppressed the tumorigenicity in colorectal cancer cells and xenograft growth in mice *via* the WNT/β-catenin pathway, which indicates an oncogenic role of YTHDF1 in CRC (69). Additionally, high levels of YTHDC2 were detected in colon tumors and were positively correlated with tumor stage, indicating that YTHDC2 may act as a diagnostic marker for CRC individuals (87, 88). These studies indicate that increased m6A level may promote the progression of CRC *via* upregulating METTL3 or readers (YTHDF1 and YTHDC2).

By contrast, METTL3 also has an inhibitory effect on the tumorigenesis of CRC cells, specifically on growth, migration, and invasion *via* regulating the p38/ERK pathway. The same group further knocked out METTL3 and found that it activated p-p38 and p-ERK in CRC, whereas the use of p38 or ERK inhibitors markedly reversed the decreased expression of METTL3 induced the migration and invasion of CRC cells (51). Other studies revealed that decreased expression of METTL14 was found in both CRC patients and cell lines. Furthermore, they suggested that METTL14 targeting miR-375 could suppress several malignant behaviors of CRC through the miR-375/YAP1 pathway and the miR-375/SP1 pathway, respectively (59). Generally, a reduction in m6A writers could also inhibit CRC through regulating various pathways.

### m6A Modification in Acute Myeloid Leukemia

Acute myeloid leukemia (AML) is a deadly hematopoietic malignancy characterized by an aggressive clonal disorder of hematopoietic stem cells (HSCs), which can prevent the differentiation of myeloid cells and generate self-renewing leukemic stem cells (LSCs) and are thought to be the leading cause of disease initiation and development (89, 90). Analyzing data from TCGA, the highest expression of METTL3 correlated with a high level of m6A was identified in AML patients, which is in accordance with an AML cell line (46). Destruction of METTL3 reverses the arrest of bone marrow differentiation and growth loss, whereas overexpression of METTL3 promotes the proliferation in AML cells *via* a chromatin-based pathway to contribute to the translation of targeted oncogenes like c-MYC, BCL2, and PTEN, suggesting that METTL3 is an essential gene for AML growth (46, 47). METTL14, another component of the methyltransferase complex, also plays an oncogenic role by regulating its downstream targeted genes (e.g., MYB and MYC) in an m6A-dependent post-transcriptional manner. Interestingly, this study further revealed that METTL14 is negatively regulated by the oncogene SPI (54). In AML, WTAP was identified to promote tumorigenesis, and its overexpression was associated with poor prognosis (55). A further study showed that in the absence of METTL3, the upregulation of WTAP is not sufficient to promote the proliferation of cells, indicating that the performance of WTAP, which is currently considered to have carcinogenic functions in AML, is strictly dependent on the regulatory function of m6A (91). Collectively, the high expression level of several writers (METTL3, METTL14, and WTAP) correlates with an increased m6A level, which plays an oncogenic role in AML.

In contrast, the demethylase FTO is highly expressed in multiple subtypes of AML, especially upregulated in AML with MLL rearrangements, compared with normal controls and AML with non-MLL-rearrangements. By regulating the expression of the leukemia-related oncogenes ASB2 and RARA, FTO overexpression can downregulate the total level of m6A, promote cell growth and colony formation *in vitro*, and promote the occurrence of leukemia *in vivo* (65). Mimicking FTO deletion, an FB23-2 structure-based design dramatically suppresses the progression of both human and mice AML cell lines, which provides a promising targeted therapeutic strategy for small-molecule inhibitors as an effective treatment of AML *via* targeting oncogenic FTO (92). Paradoxically, two independent studies on ALKBH5 demonstrated different findings based on the TCGA AML data set. Ley et al. reported the deletion of ALKBH5 in AML patients, especially those with frequent *TP53* mutations, suggesting that ALKBH5 has a tumor inhibiting effect in AML (93). However, in a study by Shen et al., ALKBH5 was aberrantly overexpressed, which is closely linked to AML patients with poor prognosis. Furthermore, they revealed that ALKBH5 facilitates tumor development *in vivo* and *in vitro* by post-transcriptional regulation of its major target, the prognosis-associated oncogene TACC3 (94). Overall, these studies suggest that higher expression of m6A erasers and decreased m6A level inhibit tumorigenesis in AML. Additional studies are needed to clarify the reasons for functional differences in ALKBH5.

## m6A Modification in other Tumors

Although the dual role of m6A modification has been shown in numerous cancers, including solid and liquid tumors, there are relatively consistent effects of m6A in other tumors. Briefly, upregulation of m6A modification on targeted genes leads to increased binding and translational efficiency of onco-RNA or ribosomes, facilitating tumorigenesis. In contrast, a decreased level of m6A level on targets can also alter the normal biological function of RNA, suppressing tumor formation.

### m6A Modification Accelerates the Formation of Pancreatic Cancer

Pancreatic cancer (PC) is a kind of malignant tumor in digestive system with poor outcomes (95). Several studies focused on the specific role of m6A-related regulators in PC patients and cells. The level of m6A modification in pancreatic patients was higher than paired normal peritumoral tissues. Among several m6A modifiers, METTL3 expression was significantly elevated in tumors and was significantly linked to high N stage and pathological stage. Decreasing the expression of METTL3 can reduce the proliferation, invasion, and migration of cancerous cell lines (96). In addition, to promote tumorigenesis, METTL3 promotes chemotherapy and radiation tolerance, which has been identified as associated with mitogen-activated protein kinase (MAPK) cascades, a ubiquitin-dependent modification in pancreatic cancer (48). Another study showed that higher METTL14 levels leads to elevated levels of m6A methylation, which accelerates the growth and metastasis of PC-targeted PERP (PMP-22 associated p53 effector), which is required for tumorigenic transformation and growth of other tumors (56, 97).

Accordingly, ALKBH5 overexpression inhibits tumorigenesis and sensitizes PC cells to chemotherapy by decreasing Wnt inhibitory factor-1 (WIF-1) mRNA methylation, which promotes the expression of WIF protein and thus downregulates the Wnt pathway (57). ALKBH5 also regulates the post-transcriptional activation of PER1 by eliminating m6A in an m6A-YTHDF2-dependent manner, thus inhibiting PC. PER1 upregulation reactivates the ATM-CHK2-P53/CDC25C pathway, thereby inhibiting the growth of cells (67). Another type of eraser, FTO, was increased in both PC tissues and cells by interacting with the proto-oncogene MYC and the transcription factor bHLH to improve its stability by lowering its level of m6A (62). The high expression of FTO in pancreatic cancer patients is positively correlated with the progression of the disease. Interestingly, a dual role of YTHDF2 was found in PC cells. On one hand, it acts as an inhibitor to suppress adhesion, invasion, migration, and the epithelial-to-mesenchymal transition (EMT) of cells through YAP signaling. On the other hand, it serves as a promoter to accelerate the growth of PC cells *via* the Akt/GSK3b/CyclinD1 pathway (71). Overall, the effects of m6A modification in PC are consistent among various groups that decreased RNA methylation triggers certain changes in tumor-specific mRNA behavior and leads to alteration in tumor-related expression of proteins, thereby contributing to tumor development.

### m6A Modification Suppresses the Progression of Renal Cell Carcinoma

As the most lethal cancer in the genitourinary system, the mortality of renal cell carcinoma (RCC) continues to increase at a rate of about 1.5% to 5.9% annually (98). Clear cell RCC is the main type of RCC, accounting for about 80% of all cases (99). One study discovered that METTL3 was lower in RCC patients than in adjacent non-tumor tissues, thus serving as a tumor suppressor. Furthermore, the alteration of the EMT, adipogenesis, and PI3K-Akt-mTOR signaling pathways were found, which indicates that these pathways may participate in mediating the impact of METTL3 on RCC (100, 101). Compared with corresponding normal control tissues, a decreased m6A level in RCC samples was reported in other studies as well. Specifically, the expression of both METTL14 and RBM15B in tissues was remarkably decreased, whereas the expression of FTO was significantly increased. Meanwhile, Kaplan-Meier analysis showed that lower METTL14 expression resulted in worse OS outcomes and suppressed P2RX6 activation (52, 53), thereby inhibiting the migration and invasion of RCC by ATP-induced Ca^2+^ influx and the regulation of the phosphorylation level of the ERK1/2 and MMP9 signaling pathways (66). After analyzing a data set from TCGA, a study identified that METTL3, METTL14, and HNRNPA2B1 serve as possible prognostic biomarkers for clear cell RCC. These collective findings suggest that reduced levels of m6A modification on targeted mRNA can also affect RNA functions, thus suppressing the progression of tumor formation.

## Progress in The Application of Small Molecules Targeting m6A Regulators

Clearly, a significant imbalance in m6A regulators has been demonstrated in many tumors. Thus, cumulative studies indicate that pharmacological regulation (inhibitors and/or activators) targeting m6A regulators to reverse this dysregulation may provide new therapeutic potential and prospects for traditional anticancer drugs.

FTO inhibitors are the first small molecules that have been identified through structure-based virtual screening and biochemical analyses to target m6A regulators, such as the natural product rhein (102), meclofenamic acid (61), and fluorescein derivatives (103). However, the activity and specificity of these FTO inhibitors are relatively poor. The latest research has discovered more effective and selective FTO inhibitors. As mentioned in AML, the FB23 and FB23-2 (the inhibitors of FTO), which are designed based on the molecular structure of the protein of FTO, increase the level of m6A methylation, inhibit the proliferation of cell lines *in vitro*, and have a significant anti-leukemia therapeutic effect on patient-derived xenograft mouse models (92). Similarly, CS1 and CS2 are also the inhibitors of FTO that were screened and selected based on their docking to the catalytic pocket of FTO (104). This study not only further confirmed suppression of the immune evasion of AML through the FTO/m6A/LILRB4 axis *in vitro* and *in vivo* but also identified that these inhibitors possess therapeutic potential in treating solid tumors, including glioblastoma, breast cancer, and pancreatic cancer. Recently, a study also identified that a new FTO inhibitor, FTO-04, could disrupt glioblastoma stem cells self-renewal, which indicates that FTO-04 may act as a potential treatment for glioblastoma (105). For the other eraser, ALKBH5, there is only one study that reported that two compounds, compound 3 (2-[(1-hydroxy-2-oxo-2-phenylethyl)sulfanyl]acetic acid) and compound 6 (4-{[(furan-2-yl)methyl]amino}-1,2-diazinane-3,6-dione), identified as ALKBH5 inhibitors, could suppress the growth of three leukemia cell lines (106). Noticeably, inhibitors of FTO and ALKBH5 are effective in certain subtypes of AMLs and some solid tumors where FTO or ALKBH5 is overexpressed and promotes tumorigenesis, suggesting that these inhibitors may have promising and broad roles in cancer therapy.

Although METTL3 is a key m6A writer, there are few studies on small molecules that could regulate its aberrant expression. Karelson et al. described the first ligands which bind the METTL3-14-WTAP complex active site to activate RNA methylation by virtual screening of molecular libraries. Further, they described the binding properties and effects on enzymatic activity (107). Similarly, Caflisch et al. initially identified and structurally characterized small molecule inhibitors of METTL3 through high-throughput docking into the SAM-binding site and protein X-ray crystallography (108). However, there is a lack of research on the activity of these small molecule compounds in cells. Until the study reported by Kouzarides et al, this is the first time that study found that STM2457, inhibitor of METTL3 exerted its effects *in vivo*. They also reported the ability of STM2457 to significantly inhibit the progression of AML (109). Collectively, the inhibition or activation of METTL3 could also provide a new potential therapeutic strategy against cancer.

In general, drugs targeting DNA and histone modifications have entered clinical therapy, whereas targeted tumor therapy based on RNA epigenetics has only just begun. Research on small molecule drugs targeting the m6A regulators is relatively limited, especially on the inhibitors or activators of some regulators, such as WATP and METTL14. At the same time, we should also realize that only a thorough and complete understanding of the mechanism of action of m6A can provide a reliable theoretical basis for drug research and development.

## Summary

This review summarizes the phenomenon that RNA methylation triggers or suppresses tumor formation and the latest studies on the application of small molecules targeting m6A regulators in various types of tumors. The importance of m6A modification has gradually been discovered by increasing numbers of studies. The correlation between the level of m6A modification, expression of m6A regulators, and clinicopathological features had been shown in diverse tumors, which may provide prognostic value in these diseases. In GM, for example, seven genetic markers were identified as risk or protective genes that independently predicted the prognosis of different grades of glioma patients (73). Similarly, the phenomenon of high or low expression of m6A regulators associated with poor outcomes is present in the majority of tumors, including BC, colorectal cancer, AML, PC, and RCC (52, 53, 55, 69, 79, 96). However, it is worth noting that in the same type of tumor, the level of m6A can have opposite effects. In the same tumor, more than one m6A-related gene generally plays a role in tumor formation, and the genes involved interact with each other. Additionally, for the same gene in the same tumor, the results can be contradictory from study to study. Also, m6A modification can switch a tumor oncogene to a tumor suppressor. Thus, there remain numerous challenges in m6A RNA modification research. First, the role of m6A in several tumors is seemingly context-dependent in different subtypes. For example, in AML, the level of m6A is higher in several subtypes of AML, especially upregulated in AML with MLL rearrangements (65). Consequently, inhibitors of FTO may only suppress the progression of certain subtypes of AML. The findings indicate the potential of m6A regulator modulation as a cancer-cell-type-selective anticancer treatment. Therefore, more convincing studies are needed to further evaluate the value of m6A in human cancers clinical studies. Second, the effects of this sophisticated and complex regulatory network on tumorigenesis, and the specific function of m6A regulators can be difficult to summarize. The differential requirement for sole regulators and the interaction between regulators in different tumors needs to be further explored in the future.

Many m6A modifier genes have been indicated to play a regulatory role in tumorigenesis, such as SOX2, ZNF217, and HBXIP (37, 39, 50). One major question that remains is whether m6A regulators participate in the development of tumors in a manner dependent on m6A, just as METTL3 promotes lung cancer in an independent manner of its catalytic activity (84). Likewise, the effect of WTAP alone in AML is not enough to promote cell tumorigenesis, when there is an absence of functional METTL3, which confirmed that WTAP functions in an m6A-dependent manner (65). Generally, analyzing data sets from public databases, scientists can assess several m6A-related genes. Further analysis of the requirements of different regulators in cancers is also important. This could help to address the functions of these regulators that are dependent and independent of m6A.

Given the core role of m6A pathways in a variety of tumors, targeted RNA modification pathways offer a promising approach for the development of innovative therapies against these diseases. However, there are relatively few small molecule drug studies based on the epigenetics of m6A regulators. Because m6A modification is a double-edged sword in the development of many tumors, only a comprehensive and complete understanding of the mechanism of action of m6A can provide a reliable theoretical basis for drug development.

## Author Contributions

All authors listed have made a substantial, direct, and intellectual contribution to the work and approved it for publication.

## Conflict of Interest

The authors declare that the research was conducted in the absence of any commercial or financial relationships that could be construed as a potential conflict of interest.

## Publisher’s Note

All claims expressed in this article are solely those of the authors and do not necessarily represent those of their affiliated organizations, or those of the publisher, the editors and the reviewers. Any product that may be evaluated in this article, or claim that may be made by its manufacturer, is not guaranteed or endorsed by the publisher.
